# Progress of ERK Pathway-Modulated Natural Products for Anti-Non-Small-Cell Lung Cancer Activity

**DOI:** 10.3390/ph18091371

**Published:** 2025-09-12

**Authors:** Lin Xing, Chi Zhang, Jieying Yuan, Kai Zhu, Helena Tomás, Ruilong Sheng, Xiuwei H. Yang, Qidong Tu, Ruihua Guo

**Affiliations:** 1 College of Food Science and Technology, Shanghai Ocean University, Shanghai 201306, China; 2 CQM—Centro de Química da Madeira, Universidade da Madeira, Campus da Penteada, 9000-390 Funchal, Portugal; 3 Department of Pharmacology and Nutritional Sciences, University of Kentucky, Lexington, KY 40506-0107, USA; 4 Jiangxi Provincial Key Laboratory of Drug Design and Evaluation, School of Pharmacy, Jiangxi Science & Technology Normal University, Nanchang 330013, China; 5 Department of Marine Biopharmacology, College of Food Science and Technology, Shanghai Ocean University, Shanghai 201306, China; 6 Marine Biomedical Science and Technology Innovation Platform of Lin-gang Special Area, Shanghai 201306, China

**Keywords:** ERK signaling pathway, natural products, non-small-cell lung cancer, bioactive ingredients, molecular docking

## Abstract

In recent decades, there has been a significant increase in new lung cancer cases and deaths globally, especially in China, which hindered the extension of human life expectancy and severely threatened public health. Natural products are important and sustainable sources of new anticancer drug molecules, offering new bioactive molecules with various structures and biofunctions for new anticancer drug development, which accounted for 40% of all anticancer drugs. Natural-based compounds could inhibit cancer cell proliferation and migration through a variety of anticancer mechanisms by the modulation/regulation of multiple biotargets and cell signaling pathways. In this review, we summarized the anticancer activities of flavonoids, terpenoids, glycosides, alcohols, coumarins, saccharides, and other natural compounds that could modulate the ERK-related signaling pathway in non-small-cell lung cancer (NSCLC) cells. We further elucidated the mechanistic pathways of natural compound combinations and computationally predicted their molecular docking affinities with ERK1/ERK2 protein targets, as well as providing an outlook on current studies, with the expectation that natural compounds will play more significant roles in future antitumor chemotherapy regimens.

## 1. Introduction

Natural products are important sources of new drug molecules, providing many structurally novel active molecules for new anticancer drug development, accounting for 40% of all anticancer drugs [1]. In recent years, employing medicinal plants for the treatment of various diseases and their therapeutic effectiveness received widespread attention, with continuous advancements in (herbal) natural product extraction methods and increased biological studies [2]. In addition, a rising drug resistance occurred in cancer cells and the side effects during anticancer drug treatment escalated the demand for new and safe anticancer drugs [3]. Some natural products, such as paclitaxel, vincristine, and demethylzeylasteral [4,5,6] and their analogs, were developed for clinical antitumor applications due to their high effectiveness, low toxicity, good selectivity, in vivo efficacy, and favorable pharmacokinetics and pharmacodynamics [7]. These natural products exhibited good antitumor activities through different mechanisms of action; consequently, systematical study on the mechanisms and biotargets offered positive feedback for the development of natural product-based antitumor drugs in cancer treatment. Through mechanism/biotarget-oriented drug design, the natural product-based antitumor drugs could be further optimized to be more effective and safer than conventional chemotherapy.

Lung cancer is one of the most common malignant cancers, with a higher mortality rate than other types of cancer. Among them, non-small-cell lung cancer (NSCLC) has a wide variety of tumors, and the causes of NSCLC are mostly related to bad lifestyle habits and daily diet. The incidence of NSCLC has been continuously increasing in China, accounting for 75–80% of the lung cancers all over the world; significantly, the 5-year survival rate of NSCLC patients is less than 25% [8]. NSCLC patients are not easy to detect at the early stage of the disease, it easily induces metastasis to other tissues, and the treatment effect is not obvious in the clinic, with unique clinical manifestations, tissue biological behaviors, epidemiological characteristics, and special therapeutic response and prognosis [9]. Most of the drugs used in modern tumor therapy were chemically synthesized agents, which were often accompanied by severe side effects or toxicity on the organism and were prone to induce drug-associated DR over an extended period of time. Hence, there is a great need to obtain natural bioactive ingredients or compounds from natural (particularly plant) resources with low side effects and stable production/yield for the treatment of NSCLC. Among them, extracellular regulated protein kinase (ERK) is the key to conducting signals from surface receptors to the nucleus. The Ras-Raf-MEK-ERK pathway mediates the transcriptional activation of Elk-1, ATF, Ap-1, c-fos, and c-Jun proteins, which are involved in a variety of biological responses such as cell proliferation and differentiation, cellular morphology, cytoskeleton construction, apoptosis, and cellular carcinogenesis [10]. Meanwhile, there were many studies on the drugs targeting this pathway for lung cancer treatment. In this paper, we reviewed the anticancer activities of flavonoids, terpenoids, glycosides, alcohols, coumarins, sugars, and other natural compounds, especially focusing on their modulation/regulation effects on ERK-related signaling pathway in NSCLC.

## 2. ERK Pathway-Modulated Natural Compounds with Anti-Non-Small-Cell Lung Cancer Activity

### 2.1. Flavonoids

Flavonoids, a family of cytoprotective compounds, were widely found in dietary vegetables, plants, and fruits. Previous studies in vitro and in vivo indicated that flavonoids are unique antioxidant molecules with therapeutic effects against colorectal, breast, prostate, lung [11,12,13,14], and many other cancers. The flavonoids’ anticancer effects are mediated through the modulation of multiple signaling pathways, such as the Akt/mTOR pathway, NF-κB pathway, AMPK pathway, Ras-Raf-MEK-MAPK/ERK pathways, as well as the STAT3 and FAK signaling pathways (Table 1).

#### 2.1.1. Scutellarein

Scutellarein (**1**), a flavonoid isolated from the leaves of *Scutellaria baicalensis*, has been reported to increase blood flow and protect heart and brain health. At a concentration of 50 μM, scutellarein showed the most significant inhibitory effect on the proliferation of human lung cancer A549 cells within 24–48 h. It could decrease the expression of p-EGFR/p-ERK/p-NFκB proteins and ultimately block COX-2 production as well as the malignant feedback loop of COX-2. These results indicated that **1**, following in vivo validation, holds potential as an anticancer drug for lung cancer treatment [15].

#### 2.1.2. Scutellarin

**Figure 1:**
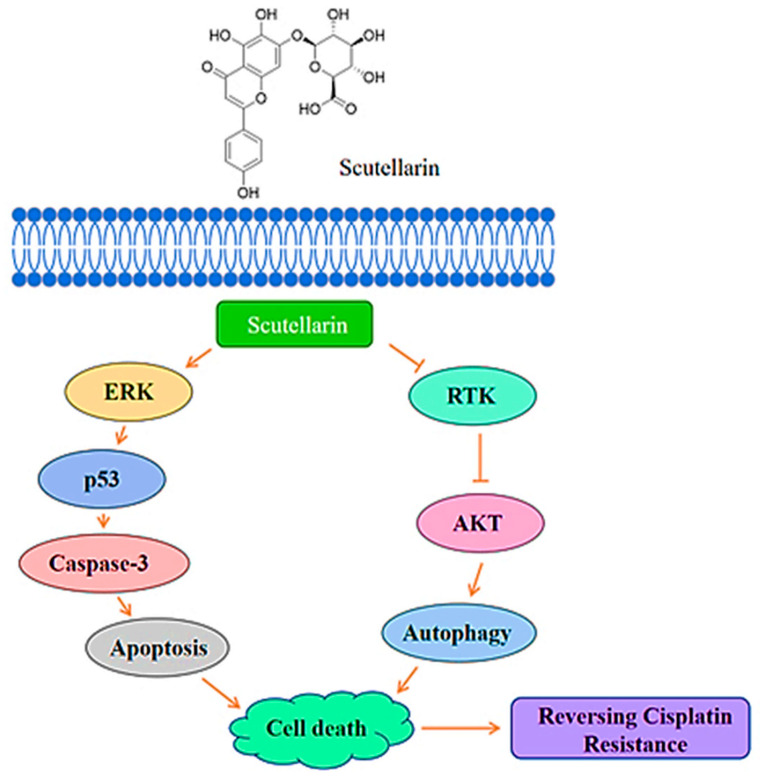
Scutellarin increases sensitivity to cisplatin in NSCLC through ERK/p53 and c-met/AKT signaling pathways.

As a glycoside derivative of scutellarein and an active flavone extracted from Chinese traditional herb *Erigeron breviscapus Hand-Mazz*, scutellarin (**2**) was generally employed for the treatment of cerebral vascular diseases. Scutellarin also had anticancer activity against various types of lung tumors and could enhance cisplatin-induced ERK activation [16]. In addition, the IC_50_ of A549 and A549/DDP cells were 0.43 and 16.07 µg/mL (0.0009 and 0.035 μM), respectively, with a resistance index of 37.37. Remarkably, the co-administration of cisplatin and scutellarin demonstrated significant synergistic effects at concentrations of 80 µM and 120 µM. Therefore, cisplatin and scutellarin could synergistically kill A549/DDP cells. Western blot assay results showed that scutellarin could enhance cisplatin-induced p53-dependent apoptosis through the activation of the ERK signaling pathway (as shown in Figure 1). Notably, scutellarin enhanced the antitumor activity of cisplatin in the PC-9 (NSCLC) cells, demonstrating that the co-treatment of cisplatin and scutellarin could synergically promote the apoptosis of lung cancer cells.

#### 2.1.3. Morusin

Morusin (**3**), a naturally occurring pentenylated flavonoid isolated from the root bark of *Morus alba* (mulberry tree), was a candidate for the treatment of lung cancers. Morusin had growth inhibitory, pro-apoptotic, and pro-autophagic effects on A549 and human lung mucoepidermoid carcinoma NCI-H292 cells. In A549 and NCI-H292 cells, the IC_50_ values of morusin treatment were 12.32 μM and 7.92 μM, respectively. Moreover, increasing the concentrations of morusin for different time intervals (up to 72 h) inhibited cell growth in a dose- and time-dependent manner. Meanwhile, morusin induced mitochondria-dependent apoptosis by causing the loss of mitochondrial membrane potential (MMP), releasing cytochrome c into cytosol, increasing the Bax level, and decreasing the Bcl-2 level. In addition, morusin inhibited the PI3K/Akt signaling pathway and further activated JNK and ERK pathways to regulate lung cancer cell survival and death [17].

#### 2.1.4. Oroxylin A

Oroxylin A (**4**) was a methylated flavonoid separated from *Scutellaria* radix, which showed obvious antitumor capability. Oroxylin A is effective in anti-metastasis in NSCLC cells in vivo and in vitro, and the related antitumor mechanisms of oroxylin A were further investigated [18]. The results showed that the oroxylin A inhibition rate (at 4, 8, and 16 μM) was around 5%, 26%, and 55% in human lung giant-cell carcinoma 95-D cells, and 11%, 36%, and 60% in A549 cells, respectively. Western blot results indicated that oroxylin A could inhibit the secretion of MMP-2 and MMP-9, and could also inhibit the expression of Snail protein by blocking the ERK/GSK-3β signaling pathway, thus eventually inhibiting lung cancer cell migration and invasion. Therefore, after in vivo experiments were completed, oroxylin A could serve as a potential therapeutic agent for treating tumor metastasis in NSCLC cells.

#### 2.1.5. Trifolium Flavonoids

Trifolium flavonoids inhibited erlotinib-resistant lung cancer PC-9R cell growth in a dose-dependent manner similar to gefitinib. Furthermore, subtoxic concentrations of trifolium flavonoids significantly increased gefitinib-induced apoptosis in PC-9R cells by cleaving PARP and caspases, reducing the expression of Bcl-2/Mcl-1. Recent investigations on the mechanism [19] disclosed that, trifolium flavonoids could significantly reduce the phosphorylation levels of STAT3 and ERK, which suggested that both STAT3 and ERK signaling pathways may be potential biotargets of trifolium flavonoids to enhance the gefitinib’s effects on NSCLC cells. The results indicated that trifolium flavonoids are chemosensitive to apoptosis in gefitinib-resistant NSCLC cells, promising its applications for the treatment of gefitinib-resistant NSCLC.

#### 2.1.6. Luteolin

Luteolin (**5**) is a widely existing dietary flavonoid that exhibited various pharmacological effects, including anti-inflammatory, antioxidant, antidiabetic, cardioprotective, and neuroprotective capabilities. The IC_50_ values of luteolin were 63.3 μM and 42.8 μM after 24 and 48 h of incubation within A549 cells, respectively [20]. Results revealed that luteolin exerted an anti-proliferation effect in a dose- and time-dependent manner in A549 lung adenocarcinoma cells, induced apoptosis with a concomitant increase in the activation of caspases-3/9, the diminution of Bcl-2, the elevation in Bax expression, and the phosphorylation of MEK and its down-stream kinase ERK, as well as the activation of Akt. Luteolin also dramatically inhibited cell motility and migration in A549 cells. The inhibitor of the MEK-ERK pathway protected against luteolin-induced cell death, and suppressed the apoptosis-inducing and anti-migratory effects of luteolin, suggesting that MEK-ERK signaling pathway plays an important role in mediating the pro-apoptotic effect and anti-migration effects of luteolin. Taken together, this study provides a new insight into the mode of action of luteolin on lung cancer.

#### 2.1.7. Chrysoeriol

Chrysoeriol (**6**) is a methylated flavonoid extracted from the tropical plant *Coronopus didymus*, and has demonstrated various biological activities such as anti-oxidation, anticancer, and anti-bacteria activities. WST-1 assay results showed that chrysoeriol has a medium antiproliferative effect in A549 lung cancer cells with an IC_50_ value of about 15 µM [21]. The mechanism study revealed that chrysoeriol exhibits a concentration-dependent inhibition of the expression of p-p38 and p-ERK1/2, which further retarded the migration and invasion of A549 cells. In vivo experiments showed that chrysoeriol (at 50 mg/kg) could inhibit the growth of xenograft cancer cells in xenografted mice models. The results indicated that chrysoeriol significantly inhibited the growth of lung cancer both in vitro and in vivo.

**Figure 2:**
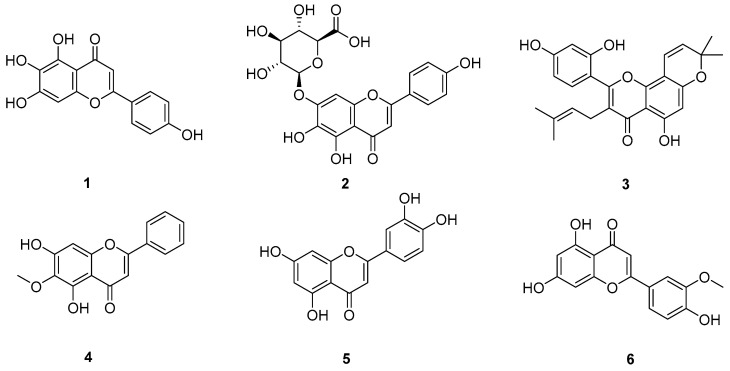
Molecular structures of flavonoids **1**–**6**: scutellarein (**1**), scutellarin (**2**), morusin (**3**), oroxylin A (**4**), luteolin (**5**), and chrysoeriol (**6**).

The structural formulas of flavonoids **1**–**6** are shown in Figure 2, and their mechanisms of action on A549 cells are summarized in Table 2.

### 2.2. Terpenoids

**Figure 3:**
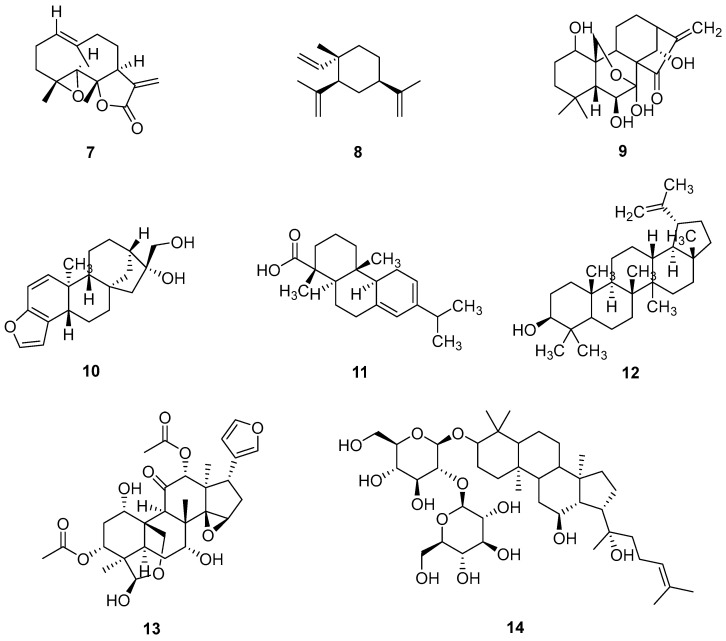
Molecular structures of terpenoids **7**–**14**: sesquiterpene lactone parthenolide (**7**), β-elemene (**8**), diterpene oridonin (**9**), kahweol (**10**), *L*-pimaric acid (**11**), lupeol (**12**), toosendanin (**13**), and 20(S)-ginsenoside Rg3 (**14**).

Terpenoids are a big family of isoprene-derived natural compounds, which widely exist in plants, fungi, bacteria, and some animals. Terpenoids play very important roles in diverse cellular processes, metabolism regulation, and pathogen-defense, and take part in many inter-species signaling and bio-interactions. Some terpenoids were employed in antitumor chemotherapy to inhibit the cancer cell division and proliferation. The structural of terpenoids **7**–**14** are shown in Figure 3, and their mechanisms of action on A549 cells are summarized in Table 3.

#### 2.2.1. Sesquiterpenoids

##### Parthenolide

Sesquiterpene lactone parthenolide (**7**) was extracted from traditional Chinese medicine Tanacetum parthenium and had antitumor activity against various tumors. Parthenolide had significant cytotoxicity of chamomile lactone against NSCLC lines GLC-82, A549, PC-9, H1650, and H1299 cells, as determined by MTT assay with an IC_50_ value ranging from 6.07 ± 0.45 to 15.38 ± 1.13 μM [22]. A study of the mechanism showed that parthenolide inhibited GLC-82 cells’ response by downregulating the expression of B-Raf, c-Myc and the phosphorylation of MEK and ERK in GLC-82 cells. Furthermore, parthenolide suppressed the development of the tumor by inhibiting STAT3 activity, which was independent of PI3K and GSK3 signaling pathways. Therefore, parthenolide can be used as a potential B-Raf/MEK/ERK pathway inhibitor to treat NSCLC.

##### β-Elemene

β-elemene (**8**) is a sesquiterpene extracted from the herb Rhizoma zedoariae. The treatment of A549 cells with β-elemene for 24 h resulted in an IC_50_ value of 50 µg/mL (0.24 µM) [23]. Thus, following treatment with 50 and 200 µg/mL for 24 h, the apoptotic rates of A549 cells increased to 10.01 ± 3.43% and 47.56 ± 4.57%, respectively. β-elemene decreased the expression of Bcl-2, increased the expression of Bax, and induced the cleavage of PARP and the phosphorylation of Akt/ERK, leading to the apoptosis of A549 cells.

#### 2.2.2. Diterpenes

##### Oridonin

Oridonin (**9**) is a diterpene compound extracted from the traditional herbal medicine Rubescensica, *Risotto japonica* and *I. trichocarpus* [24]. The traditional herbal medicine Rubescensica are Lamiaceae family plants that can be found in many regions of eastern Asia, particularly in China. Over the past few centuries, oridonin-producing plants have often been used in traditional Chinese medicine for their medicinal therapeutic properties. Modern biomedicine study disclosed that oridonin-producing plants have anti-inflammatory, anticancer, and antiviral activities. Oridonin exhibited promising anticancer activity against a wide range of types of cancer cells, it had moderate cytotoxicity against A549 and H1975 NSCLC cells with IC_50_ values of 55.91 and 15.53 µM, respectively, which indicated that oridonin has a moderate in vitro inhibitory effect on the proliferation of gefitinib-resistant NSCLC. Moreover, oridonin downregulated/inhibited the phosphorylation of EGFR and ERK significantly, suggesting that it might be inhibiting the proliferation, invasion, and migration of gefitinib-resistant NSCLC by inhibiting EGFR/ERK/MMP-12 signaling pathways, which made it a potential effective candidate to treat gefitinib-resistant NSCLC.

##### Kahweol

Coffee beans contain more than a thousand compounds, including diterpenes; kahweol (**10**) is a diterpene compound extracted from Arabica coffee beans, and it has a variety of biological activities [25]. After 48 h of treatment with different concentrations of kahweol (30, 60, and 90 µM), the lung cancer cell viability was calculated using the MTS assay. The inhibition rates of kahweol for epithelial-like lung cancer NCI-H358 cells were tested as 90.1% ± 0.02%, 84.6% ± 0.02%, and 60.6% ± 0.05%, respectively. In the case of NCI-H1299 (also known as CRL-5803) NSCLC cells, the inhibition ratios were 62.5% ± 0.01%, 40.4% ± 0.01%, and 18.4% ± 0.01%, respectively. Kahweol inhibited the expression level of BTF3 in NSCLC cell lines. Moreover, it also inhibited the ERK pathway and suppressed the inhibitors of MEK activation, causing cell cycle arrest and apoptosis. In addition, concentration-dependent and/or time-dependent inhibition constant (K_i_) measurement is still required to further understand the ERK pathway modulation capability.

##### *L*-Pimaric Acid

*L*-pimaric acid (**11**), the main component of Pine Oleoresin, has demonstrated various biological activities such as antioxidant, antibacterial, and cardiovascular effects [26,27]. The IC_50_ value of *L*-pimaric acid was 12 μM in A549 cells. *L*-pimaric acid decreased LC3 II, and increased LC3 I and p62 protein expressions, indicating that *L*-pimaric acid resulted in autophagy induction in lung cancer cells. Moreover, *L*-pimaric acid could induce the death of drug-resistant lung cancer cells by downregulating Bcl-2, upregulating Bax, activating the p38 MAPK/JNK signaling pathways, and inhibiting the ERK pathway.

#### 2.2.3. Triterpenes

##### Lupeol

Lupeol (**12**) is a triterpenoid found in many dietary fruits, vegetables and medicinal plants such as cabbage, green pepper, and strawberry. Pharmacological studies in vitro and in vivo demonstrated that lupeol had various pharmacological activities, such as anti-diabetic, anti-asthmatic, anti-inflammatory, and antitumor effects [28,29]. Transwell cell migration assay showed that in A549 cells exposed to lupeol (at 50 µM and 100 µM), the cell migration was inhibited by decreasing the expression of pERK1/2 and EMT genes, and it potently targeted ERK and MEK proteins. This suggested that lupeol might serve as an effective anti-metastatic agent candidate for lung cancer treatment after completing in vivo experiments.

##### Toosendanin

Toosendanin (**13**), a triterpenoid isolated from *Melia toosendan Sieb. et Zucc.*, was the main component of an anti-roundworm drug prescribed in China. At concentrations of 50 and 40 nM (0.05 and 0.04 μM), treatment with toosendanin for 48 h showed a slight decrease in A549 and H1975 cells’ viability, respectively [30]. Toosendanin significantly inhibited TGF-β1-induced EMT, adhesion, invasion, and the migration of A549 and H1975 cells via ERK1/2 and Snail pathways.

##### 20(S)-Ginsenoside Rg3

*Panax ginseng* (Asian ginseng) is a well-known traditional Chinese medicine with adaptogenic properties. Over the past hundred years, it was widely used by the people in China and Korea to enhance vitality, improve body health, and promote human longevity. Modern medicinal studies disclosed Panax ginseng’s potential application as a natural anticancer herb; it could inhibit tumor cells proliferation, presenting it as an attractive candidate for cancer treatment. The main active ingredient of ginseng, triterpenoid saponin 20(S)-ginsenoside Rg3 [20(S)-Rg3] (**14**), is mainly derived from the roots of ginseng. The MTT assay revealed that the A549 cell viability of 20(S)-Rg3 stabilized at approximately 50 µM; moreover, subsequent treatments at the concentrations of 24, 30, and 37 µM corresponded to IC_20_, IC_40_, and IC_60_ in A549 cells [31]. The mechanism study suggested that 20(S)-Rg3 inhibited A549 cells’ proliferation through downregulating CDK2/CyclinA2/CyclinE1, leading to the cell cycle arrest at the G0/G1 phase; moreover, the EGFR/Ras/Raf/MEK/ERK pathway was also affected by 20(S)-Rg3.

**Table 3:** Mechanism of action of terpenoids on A549 cells.

Natural Compound	Targeting Signaling Pathway Proteins	Treated Lung Cancer Cell Lines	IC_50_ Values (A549)	Ref
Parthenolide (**7**)	↓ STAT3, B-Raf, c-Myc, p-MEK, p-ERK	GLC-82, A549, PC-9, H1650, H1299	6.07 ± 0.45 to 15.38 ± 1.13 μM	[22]
β-elemene (**8**)	↑ Bax, PARP, p-Akt/ERK ↓ Bcl-2	A549	50 µg/mL (0.24 µM)	[23]
Oridonin (**9**)	↓ p-EGFR, p-ERK	A549, H1975	55.91 µM	[24]
Kahweol (**10**)	↓ BTF3, ERK, inhibitors of MEK activation	NCI-H358, NCI-H1299	No data	[25]
L-pimaric acid (**11**)	↑ Bax, LC3 I, p62, p38 MAPK/JNK, ERK ↓ LC3 II, Bcl-2	A549	12 μM	[26,27]
Lupeol (**12**)	↓ pERK1/2, EMT	A549	No data	[28,29]
Toosendanin (**13**)	↑ ERK1/2, Snail ↓ TGF-β1	A549, H1975	No data	[30]
20(S)-ginsenosideRg3 (**14**)	↓ CDK2, CyclinA2, CyclinE1	A549	No data	[31]

↑: upregulation of protein expression. ↓: Downregulation of protein expression.

### 2.3. Glycosides

**Figure 4:**
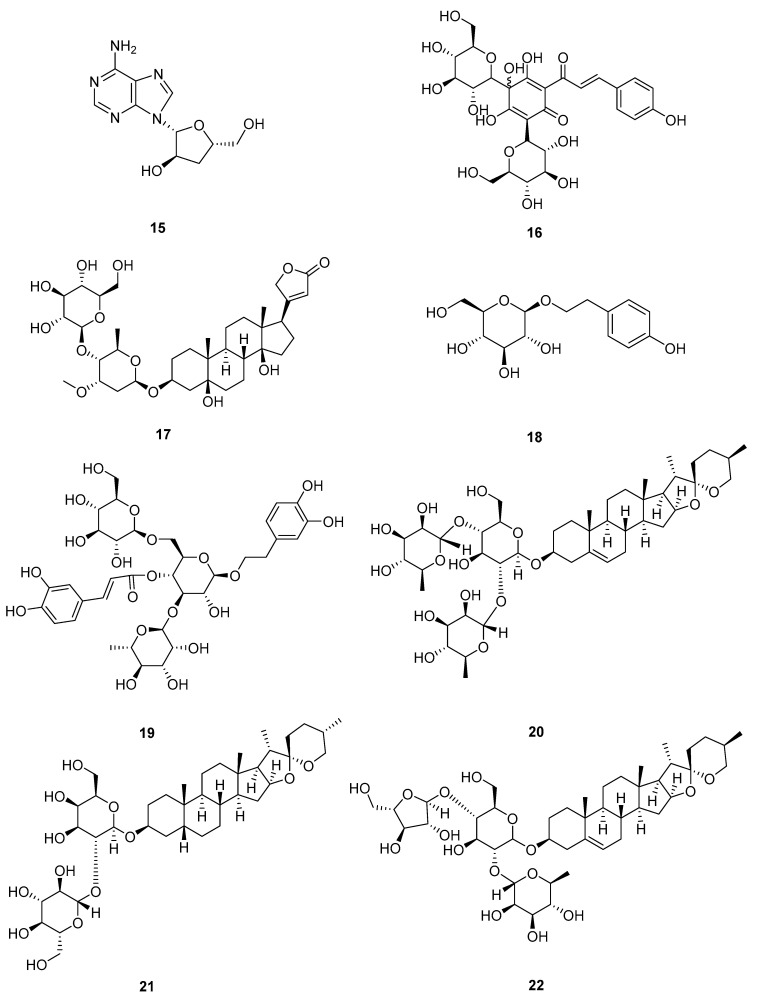
Molecular structures of glycosides **15**–**22**: cordycepin (**15**), hydroxysafflor yellow A (**16**), periplocin (**17**), salidroside (**18**), echinacoside (**19**), dioscin (**20**), timosaponin AIII (**21**), and paris saponin I (**22**).

Glycosides are a family of sugar (monosaccharide or oligosaccharide)-containing natural products; structurally, they have one or several glycones (carbohydrate part) attached to a hydrophobic molecule (aglycone, non-carbohydrate part) via a glycosidic bond. Glycosides were widely found in plants, animals, and bacteria, and are particularly rich in plants. Many glycosides were disclosed to have anticancer therapeutic effects. The structural of glycosides **15**–**22** are shown in Figure 4.

#### 2.3.1. Cordycepin

The biometabolite cordycepin (**15**) was first isolated from the fermented broth of the medicinal mushroom cordyceps militaris. Cordycepin (3′-deoxyadenosine) is a nucleoside analog that is similar to the structure of adenosine, which is reported to increase anticancer efficacy by removing the 3′-hydroxyl group and interfering with various biochemical and molecular processes [32]. Cordycepin inhibited the viability of A549 cisplatin-resistance (CR) cells in a dose-dependent manner (200, 40, 8, and 1.6 µg/mL). The IC_50_ of A549CR cells by cordycepin was determined to be 39.76 ± 10.35 µg/mL (0.16 μM). Cordycepin inhibited cells’ proliferation by downregulating the expression of H-Ras in the cells, which subsequently reduced the expression of Raf, MEK1/2, and ERK1/2 in the MAPK pathway, leading to a decrease in the viability of A549CR cells.

#### 2.3.2. Hydroxysafflor Yellow A

Hydroxysafflor yellow A (**16**), a chalcone glycoside extracted from safflower, was widely used in clinical treatment due to its antioxidation, anti-inflammation, and antitumor effects [33,34]. Upon treatment with hydroxysafflor yellow A (5, 10, and 20 μM), cell proliferation of A549 and H1299 was significantly suppressed in a dose- and time-dependent manner compared to the lipopolysaccharide (LPS) control group. In addition, LPS obviously increased the expression of Bcl-2 and decreased the levels of cleaved-caspase-3, cleaved-caspase-9, and Bax, while hydroxysafflor yellow A could significantly suppress the effect of LPS on the levels of apoptosis-related proteins in A549 and H1299 cells. Hydroxysafflor yellow A could reduce the migration and invasion induced by LPS with decreased expression of COX-2/MMP-2/MMP-9 and the mitigated release of inflammatory factors. The results indicated that hydroxysafflor yellow A suppressed LPS-mediated proliferation and induced apoptosis in A549 and H1299 cells via PI3K/Akt/mTOR and ERK/MAPK signaling pathways. While these studies offer valuable insights into the interplay between inflammatory signaling and ERK pathway activation; LPS primarily engages Toll-like receptor 4 (TLR4) signaling. This model is distinct from the canonical driver oncogenes (e.g., KRAS, EGFR) that initiate and sustain ERK signaling in NSCLC. Consequently, inferences drawn from LPS models regarding ERK pathway dynamics in NSCLC should be made with caution, as the upstream triggers and cellular context differ significantly.

#### 2.3.3. Periplocin

Periplocin (**17**) is a cardiac glycoside from Cortex Periplocae. The lung cancer inhibitory effects of periplocin were evaluated on nine lung cancer cells [35]. After 48 h of treatment, the IC_50_ values for A549, SPCA-1, H1975, NCI-H446, NCI-H460, NCI-H292, NCI-H69, 95D, and LL/2 cells were determined to be 0.08, 0.24, 0.21, 0.18, 0.4, 25, 37, 0.43, and 0.35 μg/mL (0.0001 μM, 0.0003 μM, 0.0003 μM, 0.0003 μM, 0.0006 μM, 0.0359 μM, 0.0531 μM, 0.0006 μM, and 0.0005 μM), respectively. The effects of periplocin on xenografted tumors in mice were investigated using the established A549 and LL/2 tumor models. The group that received 50 μg of periplocin showed a significantly prolonged survival time. Meanwhile, periplocin caused cell cycle arrest in G0/G1 phases, which led to apoptosis in cancer cells. Further studies showed that its induced apoptosis was associated with the inhibition of Bcl-2 and the activation of Bax. In addition, periplocin could inhibit the growth of lung cancer both in vitro and in vivo, which could be attributed to the inhibition of proliferation and the induction of an apoptosis signaling pathway, such as AKT and ERK.

#### 2.3.4. Salidroside

Salidroside (**18**) is a phenylethanoid glycoside extracted from dried roots, stems, or whole grass of *Rhodiola rosea*, exhibiting various biological activities including antidepressant, anti-aging, antiviral, anti-inflammatory, anti-hypoxic activity, and the prevention of altitude sickness [36,37,38,39,40,41,42]. Salidroside could inhibit the proliferation of A549 cells in a dose-dependent manner with an IC_50_ value of 6.2 μM. Western blot analysis showed that salidroside induced apoptosis by decreasing the expression of Bcl-2 and increasing the expression of Bax, caspase-3, and caspase-9. In addition, salidroside inhibited cell migration and invasion by decreasing the expression of active-MMP2/RhoA/ROCK1. Further studies showed that salidroside inactivated AKT and blocked the MEK/ERK signaling pathway. The dose and time-dependent modulation of AKT and MEK/ERK signaling pathways of salidroside needs to be further investigated.

#### 2.3.5. Echinacoside

Echinacoside (**19**) is one of the main phenylethanoid glycosides isolated and purified from Cistanche tubulosa, which was found to have an inhibitory effect on aging and fatigue [43]. Echinacoside inhibited the proliferation of A549 and H1299 cell lines with IC_50_ values of 45.35 μM and 68.74 μM. Moreover, echinacoside inhibited p-Raf, p-MEK1/2, and p-ERK1/2 proteins by decreasing the expression of c-Myc and c-Fos, leading to the inhibition of the Raf/MEK/ERK signaling pathway and the induction of cells apoptosis. Simultaneously, the classic A549 nude mice model of tumor formation was employed to evaluate the efficiency of echinacoside intervention treatment.

It could be noted that the compounds (**16**), (**18**), and (**19**), which were phenylethanol or chalcone glycosides with various numbers of glycoside moieties, showed differences in signaling pathway interactions within A549 lung cancer cell lines (Table 4).

#### 2.3.6. Dioscin

Dioscin (**20**) is a phytosteroidal saponin isolated from some medicinal plants, such as *Dioscorea nipponica* Makino and *Dioscorea zingiberensis* Wright. It had anti-inflammatory, lipid-lowering, anticancer, and hepatoprotective effects [44,45,46,47,48,49]. The effect of dioscin on the cell proliferation of A549 and H1299 cells was determined by MTT cytotoxicity assay; the cells were treated with different concentrations of dioscin (0–5 μM). The results indicated that dioscin inhibited the lung cancer cell growth in a dose-dependent and time-dependent manner. Dioscin blocked cell proliferation by increasing the number of cells in the sub-G1 phase. Furthermore, dioscin regulated PI3K/Akt/mTOR/ERK1/JNK1 signaling pathways by increasing the expression of ERK1/2 and JNK1/2 as well as decreasing the expression of PI3K/Akt/mTOR phosphorylation, leading to cell autophagy.

#### 2.3.7. Timosaponin AIII

Timosaponin AIII (**21**), a type of steroidal saponins isolated from *Anemarrhena asphodeloides*, exhibited the capability to improve learning and memory deficits through anti-inflammatory effects. CCK-8 assay was used to evaluate A549 cell viability: the cells were treated with increasing dosages of timosaponin AIII (0, 3, 4, 5, 6, 10, 15 and 30 μM), and the IC_50_ was determined to be 10 μM [50]. Timosaponin AIII inhibited the cell cycle at the G0/G1 phase, decreased p-ERK1/2, p-c-Scr, and p-FAK, and induced early and late apoptosis through ERK1/2 and SrcFAK signaling pathways, respectively. Further study showed that timosaponin AIII inhibited MMP-2 and MMP-9 by suppressing their mRNA levels and inhibiting β-linker protein signaling, which in turn affected the migration and invasion of cancer cells.

#### 2.3.8. Paris Saponin I

Paris saponin I (**22**) is a steroidal saponin isolated from *Paris polyphylla*, a plant species belonging to the genus *Paris* within the family Melanthiaceae. *Paris polyphylla* was used in traditional Chinese medicine for its analgesic effect. The paris saponin I exhibited enhanced anticancer activity when combining with broad spectrum antitumor compounds camptothecin (CPT) and 10-hydroxycamptothecin (HCPT) [51]. Apoptosis experiments showed that compared to CPT/HCPT acting alone, the combination of paris saponin I with CPT/HCPT promoted apoptosis in H1299 and H446 cells, indicating a synergistic effect of paris saponin I combined with CPT/HCPT. Furthermore, the combination of CPT/HCPT and paris saponin I could synergistically enhance apoptosis in H446 cells by upregulating the expression levels of cytochrome C, cleaved PARP, and Bax, along with the improved activity of caspase 3/7. It resulted in the downregulation of anti-apoptotic proteins Bcl-2 and Bcl-xl. In addition, the combination of paris saponin I and CPT/HCPT increased the expression of p-p38 MAPK in H1299 cells and the expression of JNK in H460 cells. These results indicated that the co-treatment of paris saponin I and CPT/HCPT regulated different signaling pathways in NSCLC and SCLC, with synergistic effects on the inhibition of lung cancer cell proliferation and inducing apoptosis.

#### 2.3.9. DT-13

DT-13, a saponin monomer isolated from the roots of *Liriope muscari*, demonstrated anti-inflammatory and antithrombotic activities, along with inhibitory effects on tumor metastasis and angiogenesis [52,53,54,55,56]. Synergistic anticancer effects could be achieved when combining DT-13 with the clinical investigational drug Vinorelbine (NVB) in the treatment of lung cancer. Compared to NVB alone, the combination of DT-13 and NVB significantly promoted the inhibitory effect of NVB on NCI-H1299 cells, with IC_50_ values decreasing from 315.07 nM (0.315 µM) to 5.37 nM (0.005 µM). In addition, the combination of DT-13 and NVB could cause cell cycle arrest at the M phase by increasing the protein levels of cyclinB1 and p-H3 (Ser10) as well as decreasing the p-cdc2. Moreover, the combined action of DT-13 and NVB activated the MAPK signaling pathway by upregulating the levels of p-ERK/p-JNK/p-p38.

### 2.4. Alcohols

#### 2.4.1. Hinesol

Hinesol (**23**), the major component of essential oil of *Atractylodes lancea* (Thunb.), possesses the most promising anticancer function. It has been commonly used in the clinical treatment of digestive disorders such as the loss of appetite, bloating and diarrhea [57]. The percentage of apoptotic cells increased to 21.2 ± 0.96% and 36.0 ± 1.04% at 2 (0.009 µM) and 8 μg/mL (0.04 µM) of hinesol treatment, respectively. Hinesol effectively inhibited the proliferation of A549 cells in a dose-dependent and time-dependent manner. In addition, hinesol induced apoptosis by increasing Bax and decreasing Bcl-2 proteins in A549 cells. Meanwhile, hinesol blocked the cell cycle at the G0/G1 phase by downregulating cyclin D1. Hinesol decreased the expression levels of p-ERK1/2 and p-MEK1/2, and reduced p-IκBα and nuclear-localized p-p65 to block the NF-κB pathway. Therefore, hinesol warrants further investigation as a promising candidate for NSCLC anticancer drug development.

#### 2.4.2. Fucosterol

Fucosterol (**24**), an important phytosterol, has antiproliferative effects on cancer cells. The IC_50_ values of fucosterol against various human lung cancer cells ranged from 15 to 60 µM. The highest IC_50_ was 60 µM against the HCC827 cell line [58]. Furthermore, fucosterol induced apoptosis in A549 and SK-LU-1 cells by increasing the expression of Bax and cleaved caspase-3. Meanwhile, differently from hinesol (**23**), fucosterol blocked the cell cycle at the G2/M phase, which reduced Cdc2/Cyclin A/Cyclin B1 protein expression. In addition, fucosterol downregulated the expression of p-Raf-1, p-MEK1/2, and p-ERK1/2, leading to the blocking of the Raf/MEK/ERK signaling pathway. In vivo results showed that fucosterol caused a significant decrease in the expression of Ki-67 (a biomarker of cell proliferation) and an increase in the level of cleaved caspase 3, significantly suppressing tumor weight and volume.

**Figure 5:**
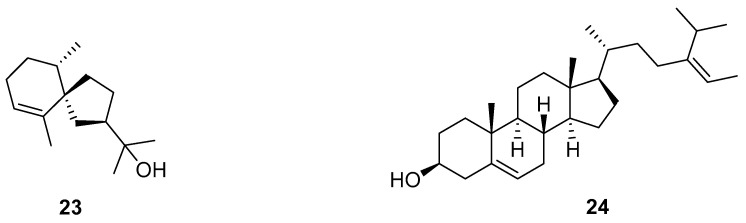
Molecular structures of alcohols **23** and **24**: hinesol (**23**) and fucosterol (**24**).

The structural of alcohols **23**–**24** are shown in Figure 5.

### 2.5. Coumarins and Their Derivatives

#### 2.5.1. 7,8-Dihydroxy-4-methylcoumarin

Coumarin was found in a variety of plants, including beans, lavender, sweet clover grass, licorice, and other edible plants such as strawberries, apricots, cherries, and cinnamon. Coumarin and related compounds have exhibited many biological activities, such as antithrombotic, antimicrobial, and anti-tumorigenic activities [59,60,61,62,63,64,65]. Using MTT assay, Goel et al. found that after 24 h of treatment with 7,8-dihydroxy-4-methylcoumarin (**25**), the IC_50_ was 160 µg/mL (0.83 µM) for A549, 180 µg/mL (0.94 µM) for HeLa, and >200 µg/mL for Hep2 and HepG2. 7,8-dihydroxy-4-methylcoumarin induced apoptosis in A549 cells through a mechanism of mitochondria-mediated activation of caspase-9/-3 and the release of mitochondrial cytochrome c. In addition, Western blot data showed that 7,8-dihydroxy-4-methylcoumarin induced apoptosis by partially inhibiting the ERK/MAPK signaling pathway.

#### 2.5.2. Angelicin

**Figure 6:**
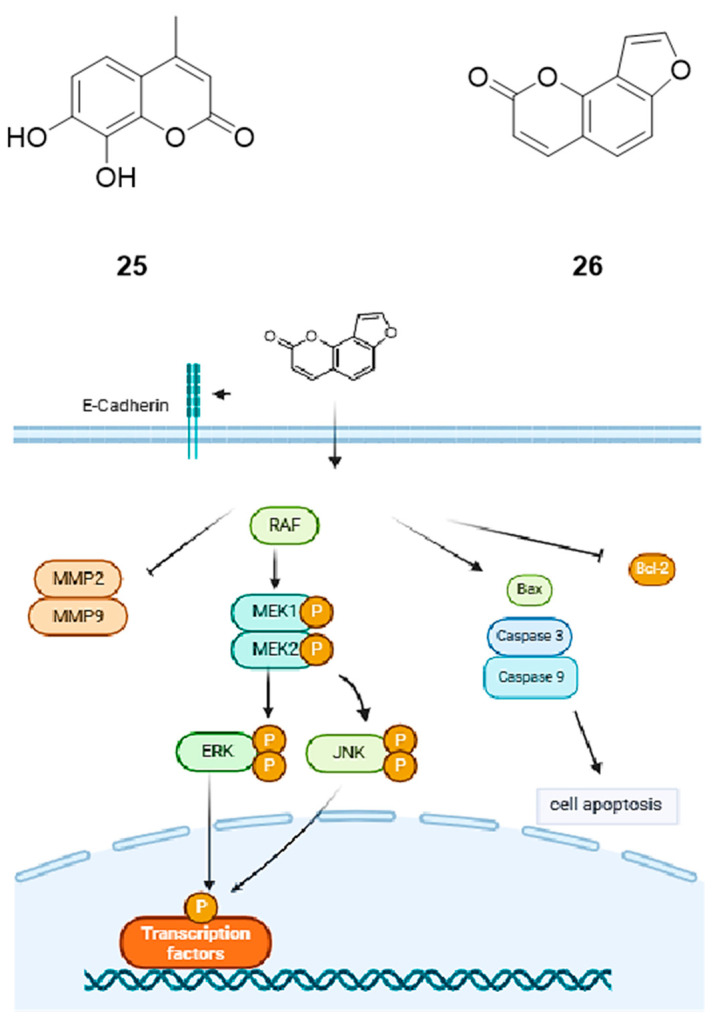
Molecular structures of coumarins: 7,8-dihydroxy-4-methylcoumarin (**25**) and angelicin (**26**) through ERK/JNK and Bax/Caspase3/9 signaling pathways.

Angelicin (**26**) is a well-known furocoumarin that has been used to treat various skin diseases. In addition, previous studies had shown that angelicin has the potential to treat leukemia by inhibiting tumor cell proliferation [66]. Angelicin inhibited the growth of A549 cells with an IC_50_ of 50.14 µM. In addition, angelicin induced apoptosis in A549 cells by increasing the expression levels of Bax/caspase-3/caspase-9 and decreasing the expression of Bcl-2. Using a nude mouse xenograft model, it was found that angelicin inhibited the migration and invasion of A549 cells by increasing the expression of E-cadherin and decreasing the expression of MMP2 and MMP9, as well as increasing the levels of p-ERK and p-JNK (Figure 6).

The mechanism of action of the above compounds on A549 cells is summarized in Table 5.

#### 2.5.3. Osthole Derivative NBM-T-BMX-OS01

Osthole (7-methoxy-8-prenyloxycoumarin) is an active compound isolated from the fruit of *Cnidium monnieri*. NBM-T-BMX-OS01(BMX), a lactone ring-opening derivative of osthole, has exhibited significant anticancer activity in combination with cisplatin [67]. The results of MTT assay showed that A549 cells treated with BMX (even at a low concentration of 0.5 µM) significantly decreased cell viability compared to cisplatin treatment alone. The results of fluorescence staining showed that the combined effect of BMX and cisplatin was due to oxidative stress induced by mitochondrial ROS generation. In addition, BMX inhibited the proliferation and colony formation of cisplatin-treated A549 cells by decreasing the phosphorylation of ERK/Akt. Based on the promising results, comprehensive investigations on the pharmacokinetics/pharmacodynamics, cell cycle arrest, other key proteins (such as Raf, MEK, p-JNK and MAPK) involved in the ERK/Akt signaling pathways, and the synergistic effect of BMX-based drug combinations are highly demanded.

**Table 5:** Mechanism of action of coumarins on A549 cells.

Natural Compound	Targeting Signaling Pathway Proteins	Treated Lung Cancer Cell Lines	IC_50_ Values (A549)	Ref
7,8-dihydroxy-4-methylcoumarin (**25**)	↑ caspase-9/-3 ↓ ERK/MAPK	A549, HeLa, Hep2, HepG2	160 µg/mL (0.83 µM)	[59,60,61,62,63,64,65]
Angelicin (**26**)	↑ Bax/caspase-3/caspase-9, E-cadherin, p-ERK, p-JNK ↓ Bcl-2, MMP2, MMP9	A549	50.14 µM	[66]

↑: upregulation of protein expression. ↓: Downregulation of protein expression.

### 2.6. Sugar/Monosaccharide

#### Mannose

Mannose (**27**) is a kind of monosaccharide that has been shown to have antiproliferative effects on a variety of cancer cells in in vitro studies [68]. Mannose inhibited the proliferation of A549 and H1299 cells in a time- and dose-dependent manner in vitro with an IC50 value of 30 mM (30,000 µM). The treatment with 30 mM mannose or 30 µM cisplatin for 24 h downregulated Bcl-2 expression in both A549 and H1299 cells in vitro. in contrast, the expression of both cleaved caspase-3 and Bax in nSclc cells was upregulated. Furthermore, the effects of mannose on the expression of MMP2, E-cadherin and N-cadherin significantly increased in a concentration-dependent manner. Mannose may inhibit the metastatic ability of nSclc cells by inhibiting EMT. It was found that treatment with 30 or 60 mM mannose for 24 h decreased the levels of p-AKT (Ser473) and p-ERK1/2 in both A549 and H1299 cells in vitro. The ratios of p-AKT/total AKT and p-ERK1/2/total ERK were significantly decreased in A549 and H1299 cells. The inhibitory regulation of the AKT and ERK signalling pathways by mannose increased in a concentration-dependent manner. Mannose may exert anticancer effects on A549 and H1299 cells by inhibiting the PI3K/AKT and ERK signalling pathways.

**Figure 7:**
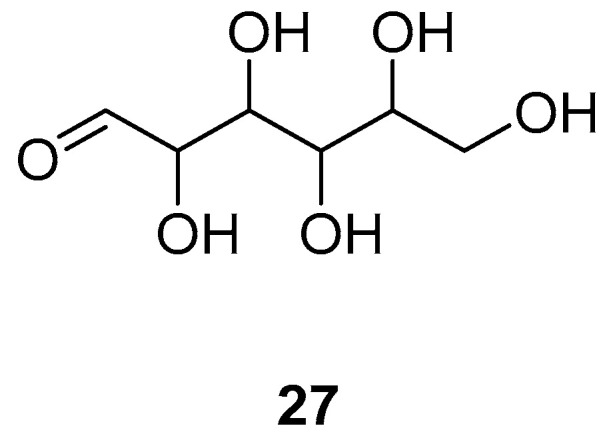
Molecular structure of sugar: mannose (**27**).

The structural is shown in Figure 7.

### 2.7. Others

#### 2.7.1. Ganoderan B

Ganoderan is one of the constituents of *Ganoderma lucidum*. Ganoderan A (GDNA), Ganoderan B (GDNB), and Ganoderan C (GDNC) are three polysaccharides isolated from the Ganoderma lucidum fruiting body [69]. GDNB (**28**) inhibited the growth of H510A and A549 cells by suppressing the expression of ki67 and PCNA. In addition, GDNB promoted the apoptosis of H510A and A549 cells by regulating the expression of Bcl-2, Bax, cleaved caspase3, and cleaved PARP. In vivo studies demonstrated that GDNB administration (10, 20, 30 mg/kg) dose-dependently reduced tumor size and weight in tumor-bearing mice compared to controls. Western blot further demonstrated that GDNB downregulated the levels of N-cadherin, vimentin, and Snail in H510A and A549 cells, while it upregulated the level of E-cadherin.

#### 2.7.2. Cinnamomum Cassia Extracts

Cinnamomum cassia is a common food spice and presents medicinal properties, such as antiviral, antioxidant, and anti-tumorigenic activities [70,71,72,73]. Cinnamomum cassia extract 60 μg/mL treatment significantly attenuated cell migration in A549 and H1299 cells. Cinnamomum cassia extracts inhibited the metastasis of A549 and H1299 cells by inhibiting the level of p-ERK1/2/FAK and downregulating the activity of MMP-2/u-PA. These findings provided useful information for future clinical trials of lung adenocarcinoma chemotherapy. Moreover, it is important to investigate the signaling pathway modulating mechanisms of the individual bioactive compounds extracted from the Cinnamomum cassia.

#### 2.7.3. Atractylenolide-1

Atractylodes macrocephala Koidz is an important ingredient in traditional Chinese herbs. One major bioactive compound, atractylenolide-1 (**29**) was reported to have anti-inflammatory and antitumor activities [74,75,76,77,78]. In A549 and H1299 cells, the IC_50_ of atractylenolide-1 action from 24 h to 72 h were 149, 143, 110, and 107, 102, and 83 μM, respectively. Atractylenolide-1 inhibited the growth of A549 and H1299 cells through the ERK1/2-mediated suppression of Stat3 and SP1 protein expressions, which subsequently reduced PDK1 gene expression. Moreover, in vivo results correspond to the in vitro findings, further confirming the inhibitory effect of atractylenolide-1 on lung tumor growth. The doses of atractylenolide-1 used in this study were based on previous studies, which showed significant inhibitory effects on tumor growth without noticeable toxicity [79].

#### 2.7.4. Solamargine

Solamargine (**30**) is a natural component of solanum lycocarpum fruit glycoalkaloid extract, and has demonstrated antitumor properties in several cancer types [80,81,82,83,84,85]. By evaluating the cell cycle in H1299 cells, solamargine was found to significantly increase the proportion of cells at the G0/G1 phase, while reducing the proportion of cells at the S phase at a concentration of 6 μM. Western blot assay showed that the high-dose solamargine treatment group significantly reduced the expression of EP4, DNMT1, and c-Jun protein expression and induced p-ERK1/2. In vivo experiments in mice revealed that mice treated with high doses of solamargine showed a significant delay in tumor growth without any severe adverse events. In addition, tumor weights and sizes were significantly reduced in the high-dose solamargine treatment group compared to the control group.

#### 2.7.5. Thymoquinone

Thymoquinone (**31**), the main bioactive component of *Nigella sativa Linn* seed oil, can be used as an anti-inflammatory, antioxidant, and anti-neoplastic agent [86,87]. Thymoquinone had a significant effect on the proliferation of A549 cells with an IC_50_ of 40 μM. The study confirmed that thymoquinone could inhibit A549 cell proliferation, migration, and invasion through the suppression of the ERK1/2 signaling pathway to inhibit MMP2 and MMP9 expression and activities.

#### 2.7.6. Sinomenine

Sinomenine (**32**), a bioactive ingredient extracted from a Chinese medicinal plant *Sinomenium acutum Rehd. et Wils*, exhibits a wide range of actions, including anti-inflammation and immunosuppression [88,89]. Human large-cell lung cancer NCI-H460 cells were treated with 200 µg/mL (0.61 μM) of sinomenine, resulting in a significant loss of cell viability as confirmed by MTT assay [90]. This showed that the activation of the PI3K/Akt/ERK signaling pathway antagonizes sinomenine-induced lung cancer cell apoptosis, and molecules that inhibit these pathways were expected to further improve the inhibitory effects of sinomenine.

#### 2.7.7. Rosemary Extract

Rosemary extracts have been shown to exhibit anticancer properties, including the inhibition of cancer cell proliferation and survival, and enhanced apoptotic activity in the colon, breast, prostate, and in leukemia [91,92]. Rosemary contains as many as 57 polyphenolic compounds, including rosmarinic acid (RA), carnosic acid (CA), and carnosol (COH). Rosemary extract inhibited proliferation significantly at 2.5 µg/mL. The IC_50_ was 19 μg/mL for H1299 cells. In addition, rosemary extract induced apoptosis in H1299 cells by increasing the level of cleaved PARP and the activation of p-ERK/MAPK, which confirmed the anticancer properties of rosemary extract.

#### 2.7.8. Maclurin

Maclurin (**33**) is a light yellow natural organic compound extracted from *Morus alba* and *Garcinia mangostana* [93]. The application of different concentrations of maclurin (0, 3, 10, 30, 60 and 100 μM) to A549 and H1299 cells (even with prolonged exposure) did not seriously affect the viability of these cells, this result suggested that maclurin has relatively low cytotoxicity. In addition, maclurin downregulated the nuclear accumulation of β-catenin by suppressing Src/FAK and ERK signaling to activate GSK3-β. As a result, transcriptional expressions of MMP-2 and MMP-9 were significantly downregulated. Consequently, the migration and invasion of A549 cells were attenuated.

**Figure 8:**
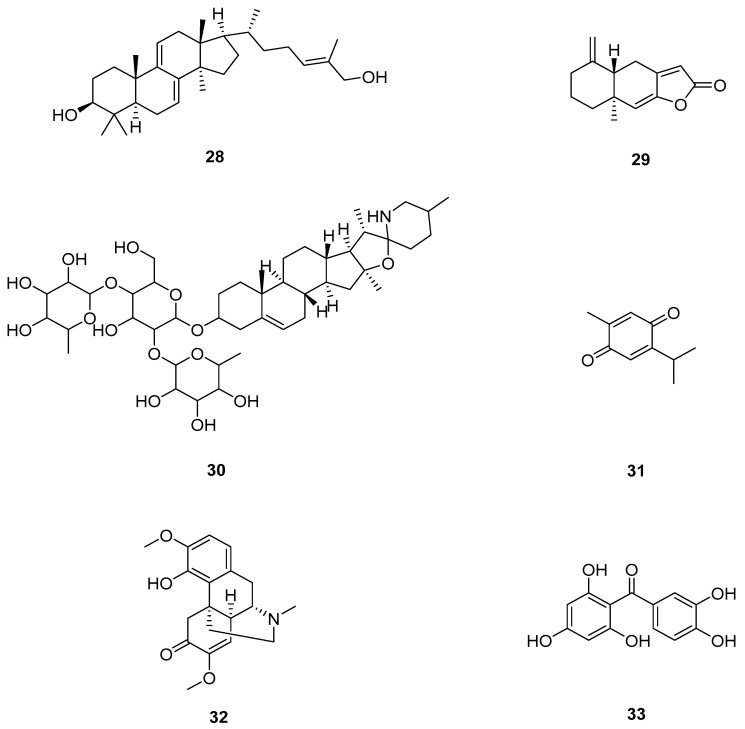
Molecular structures of compounds **28**–**33**: ganoderan B (**28**), atractylenolide-1 (**29**), solamargine (**30**), thymoquinone (**31**), sinomenine (**32**), and maclurin (**33**).

The structures of other compounds **28**–**33** are shown in Figure 8.

#### 2.7.9. Marsdenia Tenacissima

Marsdenia tenacissima is the dried cane of the Asclepiaceae plant clearance vine with activities such as use as an expectorant, for diuresis, and eliminating heat [94]. Marsdenia tenacissima extract suppressed lung cancer cell proliferation with an IC_50_ value of 92.5 ± 4.3 mg/mL in A549 cells and 82.5 ± 4.9 mg/mL in H1975 cells. Marsdenia tenacissima extract induced apoptosis in lung cancer cells by decreasing caspase-3 zymogens expression/Bcl-2 and increasing caspase-3 activities/Bax. In addition, marsdenia tenacissima disrupted autophagic flux in NSCLC cells through disrupting the fusion of autophagosome and later endosome/lysosome, and regulated apoptosis and autophagy through the MEK/ERK pathway.

## 3. Molecular Docking Study Applied to the Discovery of Anti-Non-Small-Cell Lung Cancer

The RAF-MEK-ERK signaling pathway is strongly associated with the development of different types of cancers [95]. In NSCLC, the ERK pathway is primarily abnormally activated through mutations in upstream driver genes. Targeting these upstream driver genes can indirectly inhibit the ERK pathway. Additionally, direct targeting of pathway nodes with ERK inhibitors, such as Ulixertinib, remains in the clinical trial phase [96]. Crystal structures of ERK1 (PDB ID: 4QTB) [97] and ERK2 (PDB ID: 8AOJ) [98] were obtained from the RCSB Protein Data Bank. Molecular docking was performed using AutoDock 4.2.6 software. To comprehensively map potential binding sites without preconceived bias, a blind docking approach was employed. The grid box was set to encompass the entire protein structure to allow each ligand full freedom to explore all possible binding pockets on ERK1 and ERK2. The detailed binding affinities and residues are documented in Table 6 and Table 7.

**Figure 9:**
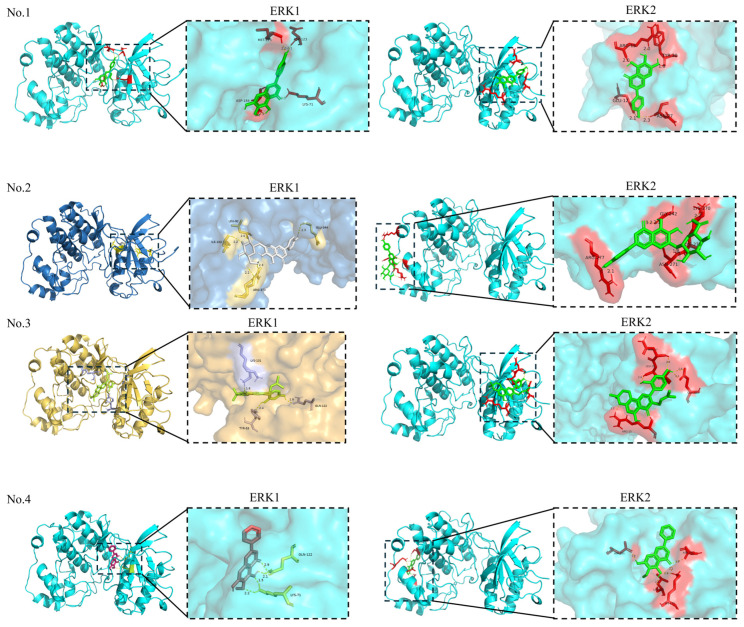

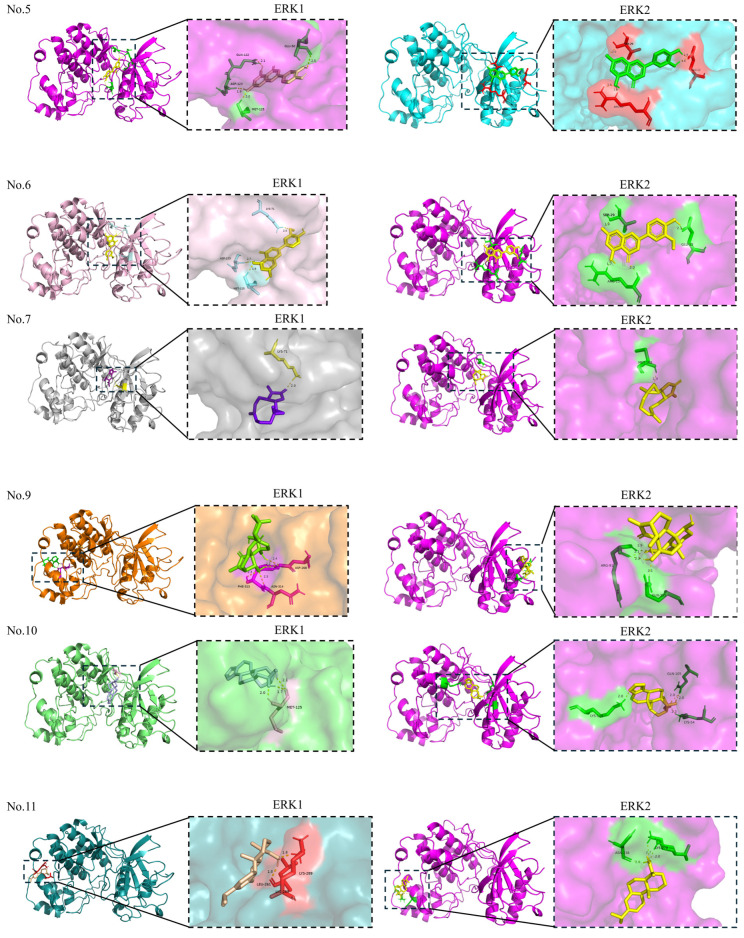

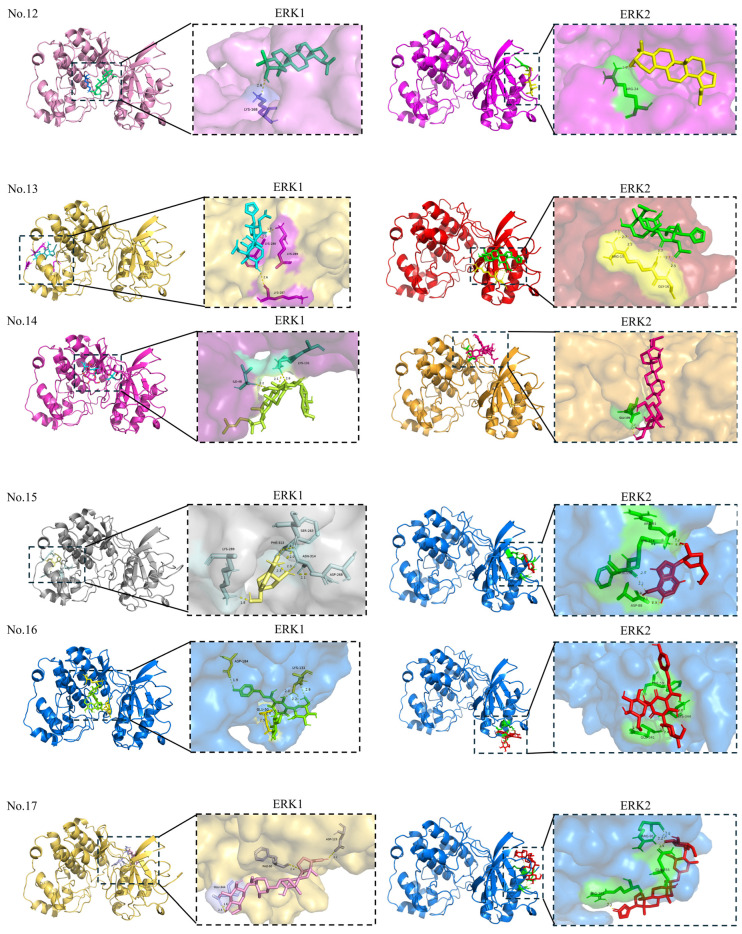

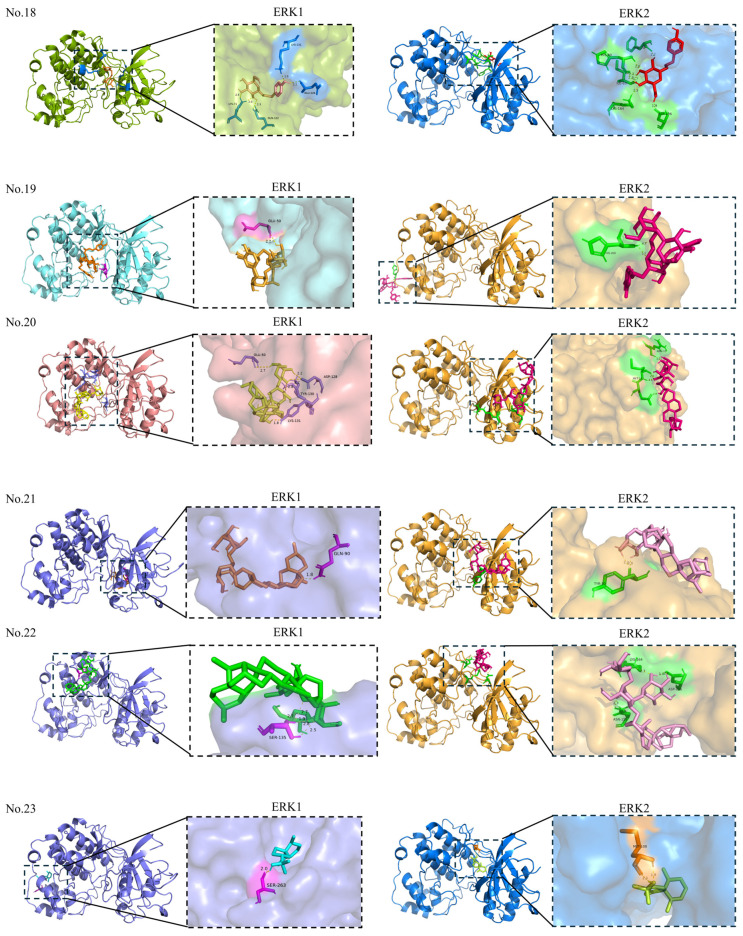

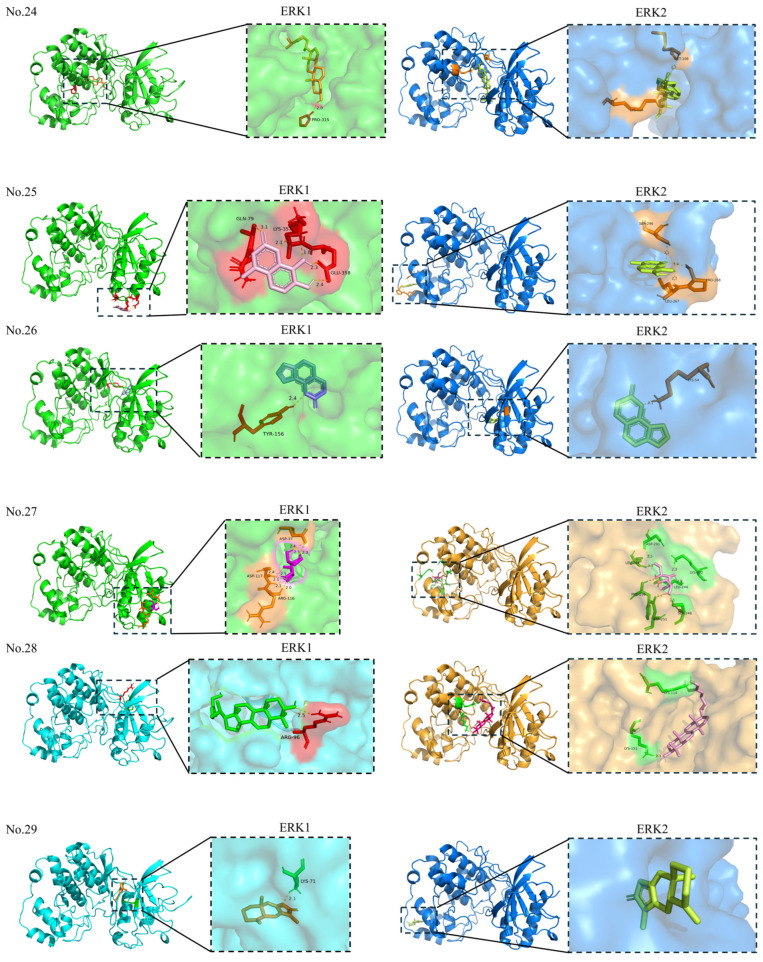

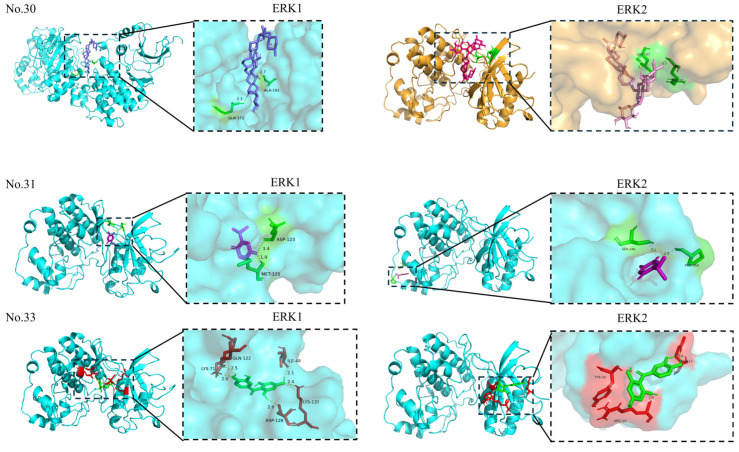
The visualization of the docking of compounds **1**–**7**, **9**–**31**, and **33** with ERK1/2.

It is generally accepted that the smaller the binding energy, the stronger the binding interaction between the ligand and the receptor. A binding energy of less than −5.0 kcal/mol suggests good binding activity. Furthermore, a binding affinity of less than −7.0 kcal/mol signifies strong binding activity [99]. The visualization of the docking of each molecule with targets is shown in Figure 9. In a molecular docking study, the binding energies between the target protein ERK1 and the active compounds ranged from −5.29 to −8.30 kcal/mol, and the binding energies between the target protein ERK2 and the active compounds ranged from −5.00 to −9.30 kcal/mol. Molecular docking confirmed strong binding between the compounds **3**, **7**, **10**, **11**, **12**, **24**, **28**, and **29** and their primary shared target ERK1, and between compounds **3**, **9**, **11**, **12**, **24**, **28**, **29**, and **30** and the target ERK2. Some compounds with binding energies greater than -5 kcal/mol may exert their biological activity not through direct binding to ERK, but by interacting with other upstream proteins that subsequently influence the ERK pathway.

**Figure 10:**
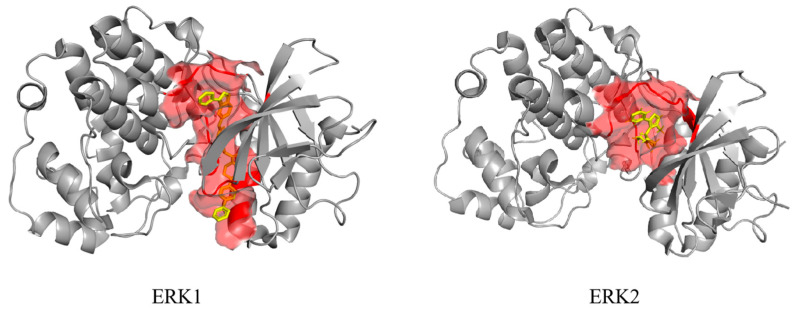
The orthosteric site range of ERK1 and ERK2.

ERK1 and ERK2 are highly homologous kinases, and their orthosteric sites correspond to the binding sites for ATP and substrate proteins within the catalytic domain. Using ligands from ERK1 (4QTB) and ERK2 (8AOJ) in the PDB database, we identified residues within a 5 Å radius as the orthosteric binding pocket (Figure 10 Red Area). Additionally, Table 6 and Table 7 classify the binding sites of the above compounds.

Based on the results of molecular docking simulations, the binding sites for the same ligand differ between ERK1 and ERK2. Wu et al. [100] identified five compounds with high ERK2 inhibitory activity through in vitro kinase assays, all exhibiting the same mechanism of action. The primary hydrogen-bonding amino acids involved are lysine (LYS-54) and methionine (MET-108). Small molecules targeting ERK2 are also likely to inhibit other kinases, reflecting the common selectivity challenge faced by kinase inhibitors. The gating site of ERK2 is glutamine (GLN-105), an amino acid appearing in less than 2% of gating sites across kinase families [101]. Therefore, during small-molecule structural optimization, introducing fragments that interact with GLN-105 may enhance compound selectivity [102]. Our molecular docking predictions indicated that **7**, **23**, and **24** bind to ERK2 at MET-108, while **10** and **26** bind at LYS-54. Additionally, **10** also formed a binding site with ERK2 at GLN-105. Therefore, further in-depth research on **10** holds greater promise as an ERK2 inhibitor.

## 4. Conclusions and Perspective

A natural product is an important source of new drugs, which provide many active molecules with novel structures for drug development. Compared to traditional chemotherapy drugs, natural products have the advantage of being renewable, inexpensive, widely available, and relatively safe. Therefore, it is necessary and meaningful to develop new natural products and enhance their anticancer activity through structural modification. This paper reviews the progress of research on the anti-lung cancer activity of compounds based on ERK-related natural product sources. It confirms the high potential of natural compounds for the treatment of non-small-cell lung cancer through the antitumor properties of flavonoids, terpenoids, glycosides, alcohols, coumarins, sugars, and other natural compounds. Developing new natural-based drugs is a lengthy and complex process, during this process, it is crucial to strictly control toxicity to normal cells and elevate the cancer/normal cell selectivity index (SI, denoted as IC_50-cancer cells_/IC_50-normal cells_) through in vivo and in vitro experiments. Moreover, using molecular diversity-oriented chemical synthetic approaches, numerous new natural product derivatives could be prepared, then by employing high-throughput screening, mathematical drug modeling and molecular docking, machine learning, and computer-aided bioinformatics to systematically study the interactions between these bioactive derivatives and key enzymes/proteins involved in the ERK-related cell signaling pathways, we could elucidate the structure–activity relationships (SAR); specifically, it is highly desirable to establish the quantitative structure–activity relationships (QSARs) for these natural ERK-pathway modulators. Generally, the QSAR formula could be expressed as bioactivity value (−LogIC_50_) = c + a_1_∗MD_1_ + a_2_∗MD_2_ + a_3_∗MD_3_ +…, where the MD_1_, MD_2_, MD_3_… are the molecular descriptors (MD) of the compounds, a_1_, a_2_, a_3_… are the corresponding coefficients, and c is the constant. Moreover, the pharmacokinetic/dynamic features including cell membrane penetration, intracellular trafficking, metabolism gateways, as well as drug-clearance and excretion need to be investigated. Therefore, it is essential for more scientists and researchers, including those studying organic chemistry, molecular biology, computational chemistry, pharmacology, and clinical medicine, to collaborate and make efforts on the development of natural product-based cell signaling pathway modulators for lung cancer treatment. In addition, we can expect that medicinal plant-based bioactive compounds will greatly improve the efficiency of chemotherapy regimens and advance clinical oncology research.

## Figures and Tables

**Table 1:** Antitumor mechanisms of flavonoids.

Diseases	Mechanisms and Pathways	In Vitro Experiment	In Vivo Experiment	Ref
Colorectal cancer	Akt/mTOR pathway, NF-κB pathway, Wnt signaling pathway, AMPK	HCT-116, HT-29 cells	Rat model. The absorption of flavonoids is 1–15% in the intestine.	[11]
Breast cancer	DNA methyltransferases (DNMTs), Histone acetyltransferases (HATs), Histone deacetylases (HDACs), Histone methyltransferases (HMTs), Histone demethylases (HDMs)	MCF-7, MDA-MB-157, MDA-MB-231 cells	Genistein did not show significant cancer-reducing activity in animal models	[12]
Prostate cancer	Fyn or Src proteins, Ras/Raf/MEK/MAPK pathways	PC3, LNCaP cells	—	[13]
Lung cancer	STAT3 and FAK signaling pathways	A549 cells	—	[14]

**Table 2:** Mechanism of action of flavonoids on A549 cells.

Natural Compound	Targeting Signaling Pathway Proteins	Treated Lung Cancer Cell Lines	IC_50_ Values (A549)	Ref
Scutellarein (**1**)	↓ p-EGFR/p-ERK/p-NFκB, COX-2	A549	50 μM	[15]
Scutellarin (**2**)	↑ ERK	A549, A549/DDP	0.43 µg/mL (0.0009 μM)	[16]
Morusin (**3**)	↑ Bax, JNK, ERK ↓ Bcl-2, PI3K/Akt	A549, NCI-H292	12.32 μM	[17]
Oroxylin A (**4**)	↓ MMP-2, MMP-9, ERK/GSK-3β	A549, human lung giant-cell carcinoma 95-D	No data	[18]
Luteolin (**5**)	↑ caspases-3/-9, Bax, p-MEK/Akt ↓ Bcl-2	A549	63.3 μM	[20]
Chrysoeriol (**6**)	↓ p-p38, p-ERK1/2	A549	15 μM	[21]

↑: upregulation of protein expression. ↓: Downregulation of protein expression.

**Table 4:** Anti-lung cancer properties and biotargets of glycosides (**16**), (**18**), and (**19**).

Natural Compound	Targeting Signaling Pathway Proteins	Treated Lung Cancer Cell Lines	IC_50_ Values (A549)	Ref
Hydroxysafflor yellow A (**16**)	↑ Bcl-2 ↓ Bax, caspase-3, caspase-9, ERK/MAPK, PI3K/Akt/mTOR, COX-2/MMP-2/MMP-9	A549, H1299	No data	[33]
Salidroside (**18**)	↑ Bax, caspase-3, caspase-9 ↓ Bcl-2, MP2/RhoA/ROCK1, MEK/ERK, AKT	A549	6.2 μM	[36]
Echinacoside (**19**)	↓ p-Raf, p-MEK1/2, p-ERK1/2, c-Myc, c-Fos	A549, H1299	45.35 μM	[43]

↑: upregulation of protein expression. ↓: Downregulation of protein expression.

**Table 6:** Docking results with ERK1 molecules in natural products.

No.	Compounds	PubChem ID	Score (kcal/mol)	Residues	Combined Site Type	Number of Hydrogen Bonds
**1**	Scutellarein	5281697	−6.81	MET-125; ASP-123; LYS-71; ASP-184	Orthosteric site	5
**2**	Scutellarin	185617	−5.59	ARG-370; ILE-103; LEU-92; GLU-344	Allosteric site	5
**3**	Morusin	5281671	−8.08	LYS-131; TYR-53; LYS-71; GLN-122	Orthosteric site	4
**4**	Oroxylin A	5320315	−6.97	LYS-71; GLN-122	Orthosteric site	4
**5**	Luteolin	5280445	−6.46	MET-125; ASP-123; GLN-122; GLU-50	Orthosteric site: MET-125; ASP-123; GLN-122 Allosteric site: GLU-50	4
**6**	Chrysoeriol	5280666	−6.62	MET-125; ASP-123; LYS-71	Orthosteric site	3
**7**	Parthenolide	7251185	−7.05	LYS-71	Orthosteric site	1
**8**	β-elemene	—	−6.03	—	—	—
**9**	Oridonin	5321010	−6.33	ASP-268; ASN-314; PHE-313	Allosteric site	4
**10**	Kahweol	114778	−7.93	MET-125	Orthosteric site	3
**11**	L-Pimaric acid	221062	−8.00	LEU-261; LYS-289	Allosteric site	2
**12**	Lupeol	259846	−8.18	LYS-168	Allosteric site	1
**13**	Toosendanin	9851101	−5.83	LYS-287; PHE-313; LYS-289	Allosteric site	3
**14**	20(S)-Ginsenoside Rg3	—	−1.47	ILE-48; LYS-131	Orthosteric site	3
**15**	Cordycepin	6303	−4.33	LYS-289; SER-263; ASP-268; ASN-314; PHE-313	Allosteric site	6
**16**	Hydroxysafflor yellow A	6443665	−2.43	ASP-184; GLU-50; LYS-131	Orthosteric site: ASP-184; LYS-131 Allosteric site: GLU-50	7
**17**	Periplocin	14463159	−5.29	GLU-344; PHE-95; ASP-123	Orthosteric site: ASP-123 Allosteric site: GLU-344; PHE-95	5
**18**	Salidroside	159278	−4.96	LYS-71; GLN-122; GLU-126; LYS-131	Orthosteric site	5
**19**	Echinacoside	—	3.93	GLU-50	Allosteric site	1
**20**	Dioscin	—	−4.18	GLU-50; LYS-131; TYR-130; ASP-128	Orthosteric site: LYS-131; ASP-128 Allosteric site: GLU-50; TYR-130	5
**21**	Timosaponin AIII	—	−2.67	GLN-90	Allosteric site	1
**22**	Paris saponin I	—	−2.83	SER-135	Allosteric site	5
**23**	Hinesol	10878761	−6.56	SER-263	Allosteric site	1
**24**	Fucosterol	5281328	−8.30	PRO-315	Allosteric site	1
**25**	7,8-Dihydroxy-4-methylcoumarin	5355836	−6.12	GLU-358; LYS-357; GLN-79	Allosteric site	5
**26**	Angelicin	10658	−6.63	TYR-156	Allosteric site	1
**27**	Mannose	—	−2.73	ARG-116; ASP-117; ASP-37	Allosteric site	8
**28**	Ganoderan B	—	−7.30	ARG-96	Allosteric site	1
**29**	Atractylenolide-1	5321018	−7.61	LYS-71	Orthosteric site	1
**30**	Solamargine	—	−6.40	ALA-191; GLN-372	Allosteric site	2
**31**	Thymoquinone	10281	−5.35	MET-125; ASP-123	Orthosteric site	2
**32**	Sinomenine	5459308	−6.16	—	—	—
**33**	Maclurin	68213	−4.67	ASP-128; LYS-131; LYS-71; GLN-122; ILE-48	Orthosteric site	5

**Table 7:** Docking results with ERK2 molecules in natural products.

No.	Compounds	PubChem ID	Score (kcal/mol)	Residues	Combined Site Type	Number of Hydrogen Bonds
**1**	Scutellarein	5281697	−6.72	ARG-15; TYR-30; GLU-12; ASN-27	Allosteric site	5
**2**	Scutellarin	185617	−5.44	ARG-277; GLY-242; ASN-271; LYS-270	Allosteric site	5
**3**	Morusin	5281671	−7.54	ARG-15; LEU-28; GLU-12; ASN-27	Allosteric site	5
**4**	Oroxylin A	5320315	−6.49	ASP-251; LEU-244; ASP-291; LYS-272	Allosteric site	5
**5**	Luteolin	5280445	−6.65	SER-29; GLU-12; ARG-15	Allosteric site	5
**6**	Chrysoeriol	5280666	−6.18	SER-29; GLU-12; ARG-15	Allosteric site	4
**7**	Parthenolide	7251185	−6.44	MET-108	Orthosteric site	1
**8**	β-elemene	—	−5.72	—	—	—
**9**	Oridonin	5321010	−7.53	ARG-91; GLN-355	Allosteric site	4
**10**	Kahweol	114778	−8.14	LYS-114; GLN-105; LYS-54	Orthosteric site	4
**11**	L-Pimaric acid	221062	−7.62	ASN-238; LYS-270	Allosteric site	3
**12**	Lupeol	259846	−8.04	ARG-24	Allosteric site	1
**13**	Toosendanin	9851101	−6.79	ARG-15; GLY-16	Allosteric site	6
**14**	20(S)-Ginsenoside Rg3	—	−3.97	GLU-109	Orthosteric site	2
**15**	Cordycepin	6303	−4.26	ARG-91; PHE-354; GLN-355; ASP-88	Allosteric site	7
**16**	Hydroxysafflor yellow A	6443665	−3.13	PHE-59; LYS-344; GLU-341	Allosteric site	5
**17**	Periplocin	14463159	−5.05	ARG-91; GLN-355; ARG-353	Allosteric site	6
**18**	Salidroside	159278	−4.58	PHE-78; HIS-80; ILE-83; LYS-164; ASP-106	Orthosteric site: ASP-106 Allosteric site: PHE-78; HIS-80; ILE-83; LYS-164	8
**19**	Echinacoside	—	−1.90	HIS-269	Allosteric site	2
**20**	Dioscin	—	−5.18	ARG-15; MET-13; GLU-12	Allosteric site	4
**21**	Timosaponin AIII	—	−5.00	TYR-30	Allosteric site	2
**22**	Paris saponin I	—	−5.12	LYS-164; ASP-106; ASN-158	Allosteric site	4
**23**	Hinesol	10878761	−6.49	MET-108	Orthosteric site	2
**24**	Fucosterol	5281328	−7.84	MET-108; LYS-114	Orthosteric site	2
**25**	7,8-Dihydroxy-4-methylcoumarin	5355836	−5.07	SER-246; PRO-268; LEU-267	Allosteric site	3
**26**	Angelicin	10658	−6.42	LYS-54	Orthosteric site	1
**27**	Mannose	—	−1.92	ASP-291; LEU-294; LYS-272; LEU-244; PHE-296; SER-248; ASP-251	Allosteric site	7
**28**	Ganoderan B	—	−7.68	LYS-114; LYS-151	Orthosteric site: LYS-114 Allosteric site: LYS-151	2
**29**	Atractylenolide-1	5321018	−7.27	—	—	—
**30**	Solamargine	—	−9.30	LEU-107; ARG-50	Orthosteric site: LEU-107 Allosteric site: ARG-50	2
**31**	Thymoquinone	10281	−5.64	SER-246; PRO-268	Allosteric site	2
**32**	Sinomenine	5459308	−6.90	LEU-107; ARG-50	Allosteric site	2
**33**	Maclurin	68213	−4.68	GLU-12; TYR-30; ARG-15	Allosteric site	4

## Data Availability

No new data were created or analyzed in this study. Data sharing is not applicable to this article.
